# Draft Genome Sequences of Four Streptomycin-Sensitive Erwinia amylovora Strains Isolated from Commercial Apple Orchards in Ohio

**DOI:** 10.1128/MRA.00893-21

**Published:** 2021-12-16

**Authors:** A. M. Jimenez Madrid, T. Klass, V. Roman-Reyna, J. Jacobs, M. L. Lewis Ivey

**Affiliations:** a Department of Plant Pathology, The Ohio State University College of Food, Agricultural, and Environmental Sciences-Wooster, Wooster, Ohio, USA; b Department of Plant Pathology, The Ohio State University, Columbus, Ohio, USA; c The Ohio State University, Infectious Disease Institute, Columbus, Ohio, USA; University of Arizona

## Abstract

Erwinia amylovora is the causative agent of fire blight, a devastating disease of apples and pears worldwide. Here, we report draft genome sequences of four streptomycin-sensitive strains of E. amylovora that were isolated from diseased apple trees in Ohio.

## ANNOUNCEMENT

Fire blight, which is caused by Erwinia amylovora, is among the most devastating bacterial diseases of apples worldwide and occurs annually in Ohio orchards. Antibiotics, especially streptomycin sulfate, are the most effective strategy to control this disease (1). However, widespread use of streptomycin has led to the emergence of streptomycin-resistant (SmR) E. amylovora strains in orchards across the United States (2). We sequenced the genomes of four streptomycin-sensitive (SmS) strains of E. amylovora that had been isolated from diseased commercial apple trees in Ohio.

Bacterial isolations from symptomatic shoots were conducted using Crosse-Goodman medium and nutrient broth yeast (NBY) agar as described previously (3). Erwinia amylovora strains (Table 1) were screened for SmR using a bioassay test (4). Single colonies were restored from 30% glycerol stocks by streaking on NBY medium, and total genomic DNA was extracted using the Nextera DNA Flex microbial colony extraction protocol (5). Extracted DNA was quantified by spectrophotometry and adjusted to 20 ng/μl for library preparation. Sequencing libraries were prepared using the Illumina DNA preparation kit, and the libraries were sequenced on the Illumina iSeq 100 platform with 150-bp paired-end sequencing. Default parameters were used for all software unless otherwise specified. Illumina Local Run Manager software was used to convert and trim the resulting sequences. The quality of sequenced reads was assessed with FastQC v0.11.9 (6). SPAdes v3.14.1 was used to *de novo* assemble the E. amylovora genomes and determine genome coverage (7). Genomes were annotated using the NCBI Prokaryotic Genome Annotation Pipeline (PGAP) v5.2 (8–10).

**TABLE 1:** Genomic information for the sequenced draft genomes of four Erwinia amylovora strains isolated from commercial apple orchards in Ohio

Species	Strain	City and county of isolation	Host	No. of reads	Genome coverage (×)	Genome size (Mb)	No. of contigs	G +C content (%)	*N*_50_ (bp)	NCBI accession no.			ANI (%) vs E. amylovora ATCC 49946	LINbase no.	LINbase best match (ANI [%])	ANI (%) vs strain:			
										GenBank	SRA	BioSample				MLI90-17	MLI90-17	MLI90-17	MLI90-17
Erwinia amylovora	MLI90-17	Wooster, Wayne County, Ohio	Apple	584,484	16	3.8	53	53.5	123,502	JAIMFV000000000	SRR16598628	SAMN20930864	99.98	51_A_0_B_0_C_1_D_0_E_0_F_0_G_0_H_0_I_1_J_0_K_0_L_0_M_0_N_1_O_0_P_0_Q_8_R_0_S_0_T_	E. amylovora NHSB01-1 (99.968)	100.00	99.90	99.989	99.90
Erwinia amylovora	MLI181-18	Lexington, Richland County, Ohio	Apple	369,454	11	3.8	63	53.5	192,887	JAIMFW000000000	SRR16598627	SAMN20930865	99.90	51_A_0_B_0_C_1_D_0_E_0_F_0_G_0_H_0_I_1_J_0_K_0_L_0_M_0_N_0_O_0_P_1_Q_1_R_0_S_0_T_	E. amylovora MAGFLF 2 (99.957)	99.90	100.00	99.894	100.00
Erwinia amylovora	MLI217-18	Laurelville, Hocking County, Ohio	Apple	387,771	15	3.8	163	53.6	172,997	JAIMFX000000000	SRR16598626	SAMN20930866	99.98	51_A_0_B_0_C_1_D_0_E_0_F_0_G_0_H_0_I_1_J_0_K_0_L_0_M_0_N_1_O_0_P_2_Q_0_R_0_S_0_T_	E. amylovora LA635 (99.943)	99.99	99.89	100	99.90
Erwinia amylovora	MLI200-18	Medina, Medina County, Ohio	Apple	270,374	10	3.8	190	53.6	156,483	JAIMFY000000000	SRR16598625	SAMN20930867	99.89	51_A_0_B_0_C_1_D_0_E_0_F_0_G_0_H_0_I_1_J_0_K_0_L_0_M_0_N_3_O_0_P_0_Q_0_R_0_S_0_T_		99.90	100.00	99.90	100

Classification of the assembled genomes was conducted by average nucleotide identity (ANI) analysis using the enveomics collection (11) and LINbase with genome sequence as the identification method (12–16). SmR in E. amylovora occurs either from the presence of *strA* and *strB* on plasmids pEA29 or pEA34 or through a mutation in codon 43 of *rpsL* (17, 18). The presence of SmR genes was analyzed by mapping strain reads to E. amylovora plasmid pEA34 (GenBank accession number M96392.1) and *rpsL* (GenBank accession number NC_013961.1) with the programs BWAv0.17 and IGVv2.10.3 and by conducting BLAST searches for these genes against the assembled genomes (19–21). The four E. amylovora strains were nearly identical to the reference strain (E. amylovora ATCC 49946 [GenBank accession number FN666575.1]), with ANI values ranging from 99.89% to 99.98% (Table 1). LINbase results confirmed E. amylovora as the best match for each sequenced genome. All four Ohio strains contained the E. amylovora strain Ea88 ubiquitous plasmid pEA29 (GenBank accession number NC_005706.1) but not *strA*, *strB*, or pEA34, indicating an SmS genotype (17, 18).

The genome sequences and genomic analysis workflow for the SmS strains provide a baseline to screen and monitor for SmR in Ohio apple orchards. Further genomic analysis of E. amylovora will increase our understanding of the genetic basis for resistance, allowing us to better address the sustainability of streptomycin use for fire blight management.

### Data availability.

Data were deposited in NCBI GenBank (BioProject accession number PRJNA756955). The partial genomes were also deposited in LINbase. The BioSample accession number, GenBank accession number, and LINbase number for each E. amylovora strain are presented in Table 1.
